# Self‐Assembly Ordered Layered Gels for Thermochromic Smart Windows With Excellent Electromagnetic Shielding Properties

**DOI:** 10.1002/advs.75518

**Published:** 2026-05-07

**Authors:** Shusheng Wang, Zijing Li, Shengchong Hui, Hongjing Wu, Shanshan Liang, Fusong Yuan, Limin Zhang

**Affiliations:** ^1^ State Key Laboratory of Porous Metal Materials School of Physical Science and Technology Northwestern Polytechnical University Xi'an China; ^2^ Center of Digital Dentistry Department of Prosthodontics Second Clinical Division National Center of Stomatology National Clinical Research Center for Oral Diseases National Engineering Research Center of Oral Biomaterials and Digital Medical Devices School and Hospital of Stomatology Beijing Key Laboratory of Digital Stomatology Research Center of Engineering and Technology for Computerized Dentistry Ministry of Health Peking University Beijing China; ^3^ Sanhang Science &Technology Building Shenzhen Research Institute of Northwestern Polytechnical University Shenzhen Guangdong China

**Keywords:** electromagnetic protection, layered gels, thermochromic smart window

## Abstract

Electromagnetic shielding materials offer excellent resistance to electromagnetic interference, yet their application remains constrained by single‐functionality in complex environments. Herein, we fabricate a novel electromagnetic shielding smart window featuring fast‐response characteristics. The proposed smart window is constructed based on a self‐assembly layered gel network, which is synthesized using two‐dimensional Laponite XLG and N‐isopropylacrylamide (NIPAM) as the matrix, coupled with functional modification via metal ions. Metal ions doped in the system effectively enhance the electrical conductivity of the material, enabling a maximum electromagnetic shielding effectiveness (SE_T_) of 72.49 dB. Furthermore, this unique layered architecture endows the system with enhanced stability and structural order, while simultaneously reducing mass transfer resistance associated with light transmission and heat conduction, thereby accelerating the responsive behavior of materials. Specifically, SCa‐1 exhibits a luminous transmittance (*T*_lum_) of 83.49% and a large solar modulation (Δ*T*_sol_) of 82.57%, which achieves a rapid thermochromic response within 20 s at 60°C. This work clarifies the role of layered structural design in optimizing system stability and order, revealing the fundamental mechanisms underlying the synergy between electromagnetic shielding and thermochromic properties, which provides a novel strategy for designing smart electromagnetic protection materials.

## Graphical abstract

Metal‐ion‐coupled self‐assembled layered gels were prepared using a simple sol–gel method, successfully integrating electromagnetic shielding and thermochromic functions into the material. SCa‐1, which exhibits the best overall performance, achieves an electromagnetic shielding efficiency of up to 72.49 dB, while maintaining a visible light transmittance (Tlum) of 83.49% and a solar radiation modulation capability (ΔTsol) of 83.05%.

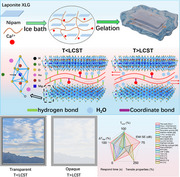

## 1 Introduction

With the rapid advancement of electronic technology, significant electromagnetic wave pollution has emerged [1, 2, 3]. To effectively address this hazard and overcome challenges posed by variable application environments, it is necessary to develop smart materials with high electromagnetic shielding performance [4, 5]. Generally, smart materials are typically driven by external stimuli such as heat, light, or electricity [6, 7, 8], exhibiting excellent adaptability and controllability. They hold excellent application potential in energy conservation, biomedicine, and flexible electronics fields [9, 10, 11]. When combined with electromagnetic shielding capabilities, they are expected to demonstrate greater application advantages. Particularly in temperature regulation and building energy efficiency, thermochromic smart windows are regarded as the next‐generation of promising devices to realize energy conservation and enhance environmental benefits [12]. It is foreseeable that smart windows with integrated electromagnetic shielding can regulate energy consumption and shield electromagnetic radiation through functional synergy. This can promote research on the integration of smart material functions and the development of new materials. Therefore, designing electromagnetic shielding smart windows with fast response speed, high energy‐saving efficiency, and excellent electromagnetic protection capabilities is crucial.

Thermochromic smart materials are broadly classified into metal oxides, perovskites, hydrated ionic polymers, and hydrogels [13, 14, 15, 16]. Among these candidates, hydrogels realize light modulation mainly via scattering state variations triggered by hydrophilic‐hydrophobic phase transitions [17]. Their high transparency and controllability make them promising for smart window applications. For gel‐based smart windows, predominant structural designs include dual networks, copolymer networks, and nanocomposite networks [18, 19, 20]. These structures have been extensively investigated, offering limited thermal response speed for innovative smart window material development. Drawing inspiration from composite gel structures incorporating two‐dimensional materials such as graphene and MXene [21], an ordered layered structure can facilitate both heat conduction and rapid responsiveness [22], significantly enhancing the thermochromic properties of the material. Furthermore, to achieve electromagnetic shielding compatibility, structural design is also widely acknowledged as an effective strategy to overcome the functional limitations of hydrogels [23]. The interfaces within the layer structure can accommodate more water molecules, benefiting the proton conduction under electromagnetic wave stimulation [24]. This effect can boost the conductivity of materials and endow them with excellent electromagnetic shielding performance. Consequently, constructing layered‐structure gels provides a promising avenue to fabricate electromagnetic shielding smart windows with excellent performance.

The selection of gel matrix and design of layered architecture must enable the material with exceptional electromagnetic shielding performance and thermochromic functionality, which meet practical application requirements. In this study, the thermochromic monomer N‐isopropylacrylamide was adopted as the gel matrix [25]. Considering that materials such as graphene and MXene are black, which restricts their use in smart windows. Therefore, the incorporation of white nano clay Laponite XLG for constructing layered architecture is an effective strategy to optimize the performance of gels [26]. Laponite XLG possesses a unique two‐dimensional lamellar morphology that can self‐assemble into the ordered arrangement within the gel network [27], thereby significantly enhancing the mechanical strength and structural stability of the composite. Most research demonstrates that the introduction of layer structure not only strengthens the capacity for reflecting and scattering electromagnetic waves but also promotes the dissipation of electromagnetic wave energy [28, 29]. Furthermore, the establishment of a layered structure facilitates the shortening of the response of Nipam segments, accelerating the thermal phase transition rate of the gel. The performance of the gel can be further enhanced via the incorporation of metal ions while retaining the structural advantages of the composite. Na^+^, Mg^2^^+^, K^+^, and Ca^2^^+^ stand out as optimal candidates owing to their inherent colorlessness. These metal ions can modulate electromagnetic shielding capability and thermochromic behavior, which exhibit excellent stability and adaptability. Specifically, coordinate bonds formed by these ions generate additional cross‐linking sites [30], thereby further enhancing the stability of the gel network. Meanwhile, non‐covalent interactions enhance the ionic conductivity [31], facilitating the shielding effect of Electromagnetic interference [32].

In conclusion, this study focuses on the fabrication and property modulation of Nipam/Laponite XLG layered composite gels coupled with metal ions. Its primary objective is to optimize properties by constructing ordered layered architectures and leveraging interactions between ions and the gel matrix, thereby addressing the limitations of Nipam‐based gels in electromagnetic shielding smart window applications, including poor mechanical performance, inadequate electromagnetic shielding efficacy, and insufficient stability. Notably, SCa‐1 attains a peak electromagnetic shielding effectiveness of 72.49 dB, while simultaneously exhibiting a luminous transmittance (*T*_lum_) of 83.49% and an outstanding solar modulation efficiency (ΔT_sol_) of 82.57%. Through a systematic investigation of the effects of metal ions on the microstructure, thermal response behavior, mechanical properties, and electromagnetic shielding performance of Laponite XLG/Nipam composite gels, this study elucidates the electromagnetic shielding and thermochromism mechanisms influenced by the layered structure. Ultimately, this work provides a theoretical foundation and technical support for the design and development of high‐performance materials for electromagnetic shielding smart windows.

## 2 Results and Discussion

### 2.1 Characterization of Layered Structure and Chemical Bonds

**FIGURE 1:**
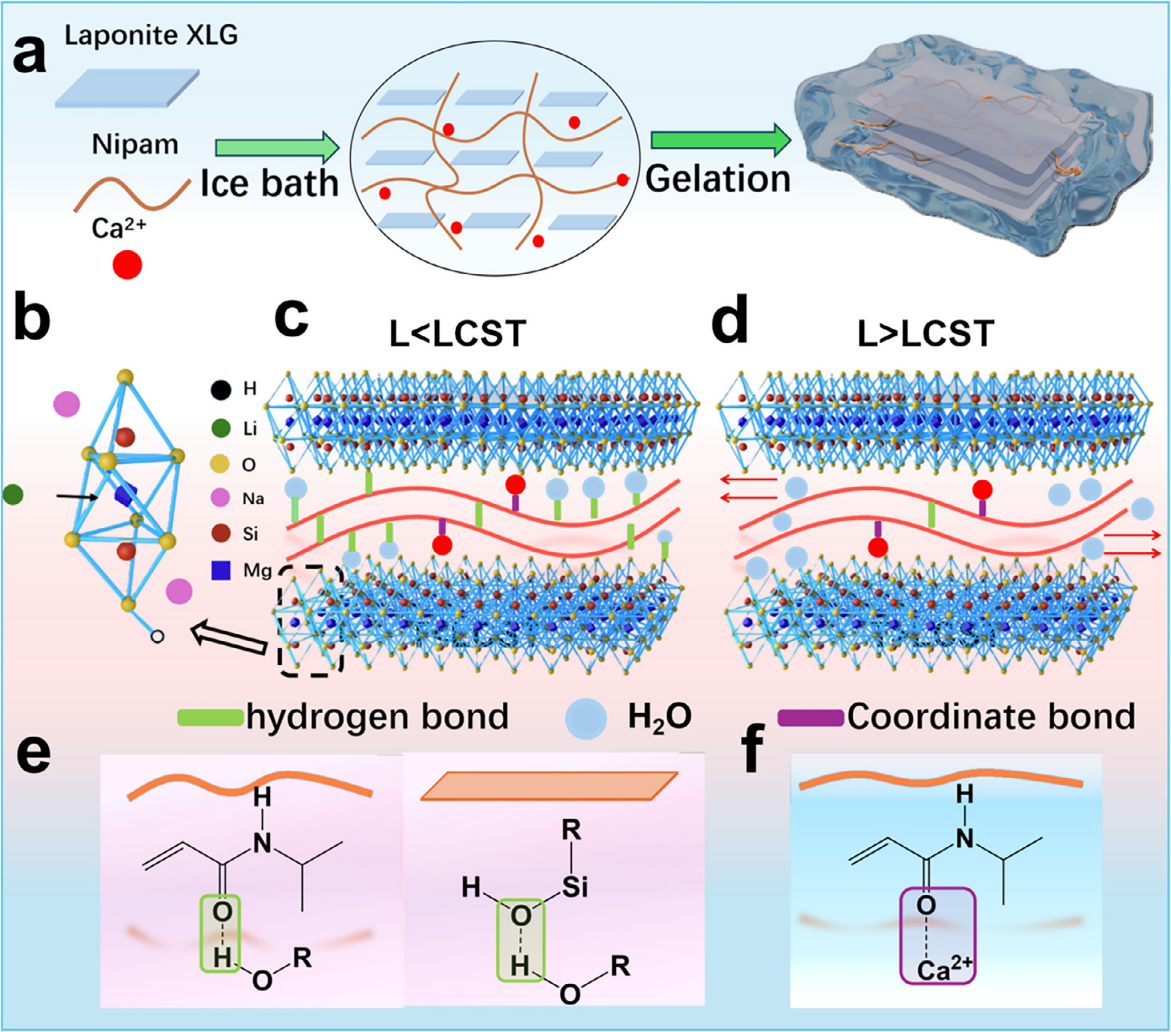
(a) The schematic illustration of the layered structure of thermochromic gels regulated by calcium ions. (b) The Microstructure of Laponite XLG. (c) Hydrophilic system and (d) Hydrophobic system of gels. (e) Hydrogen bonds and (f) Coordinated bonds within the system.

The self‐assembled layered structure composite gel is prepared through the sol–gel method, as shown in Figure 1a. Taking Laponite XLG as the matrix, a layer structure of two‐dimensional materials was constructed. Subsequently, polymer chains of Nipam were coupled to this layered structure via hydrogen bond, which facilitated the formation of a stable interactive gel network [33]. To further optimize the network performance, calcium ions were introduced to reinforce both the stability and flexibility of the system through covalent interactions. This modification strategy ultimately yielded a functional gel with more comprehensive performance attributes. Its structural characteristics are primarily illustrated in Figure 1b. Specifically, the single silicate layer features a classic sandwich‐like architecture, where the tetrahedral structures constituted by silicon and oxygen atoms function as the interlayers, and the octahedral structures composed of magnesium/lithium and oxygen atoms serve as the core layers [34]. Through the short‐range ordered arrangement of a large ensemble of atoms, these structural units further assemble into a nano‐layered configuration. Additionally, some hydroxyl groups substitute for oxygen atoms, which significantly enhances the water adsorption capacity of the material.

The thermochromism properties of the system endow it with excellent potential for application as an intelligent window. Below the lower critical solution temperature (LCST), the internal structure of the system is illustrated in Figure 1c, where the amide groups of Nipam molecular chains and the nanosheets of Laponite XLG self‐assemble into a hydrophilic homogeneous system, imparting the material with favorable transparency and electromagnetic shielding performance [35]. This ordered structure can accelerate heat transfer and enhance the response speed of materials. When the gel is exposed to temperatures exceeding the LCST, the vigorous thermal motion of water molecules drives the system to transform into a hydrophobic heterogeneous regime dominated by isopropyl groups (Figure 1d). In this state, the material exhibits light impermeability [36]. The non‐covalent interactions within the system are primarily formed by hydrogen bonds among water molecules, amide groups in Nipam, and hydroxyl groups in Laponite XLG. The ordered formation of the layered structure intertwines with the hydrogen bond network to yield a more robust system (Figure 1e), enabling the sacrificial bond to achieve energy dissipation under external stress [37]. The layered architecture maintains the gel network framework while enhancing material stability and durability. Furthermore, calcium ions, acting as a cross‐linker, coordinate with oxygen atoms in amide groups to generate additional cross‐linking sites (Figure 1f). [38] This interaction not only reinforces the stability of the material system but also endows the gel with enhanced environmental adaptability.

**FIGURE 2:**
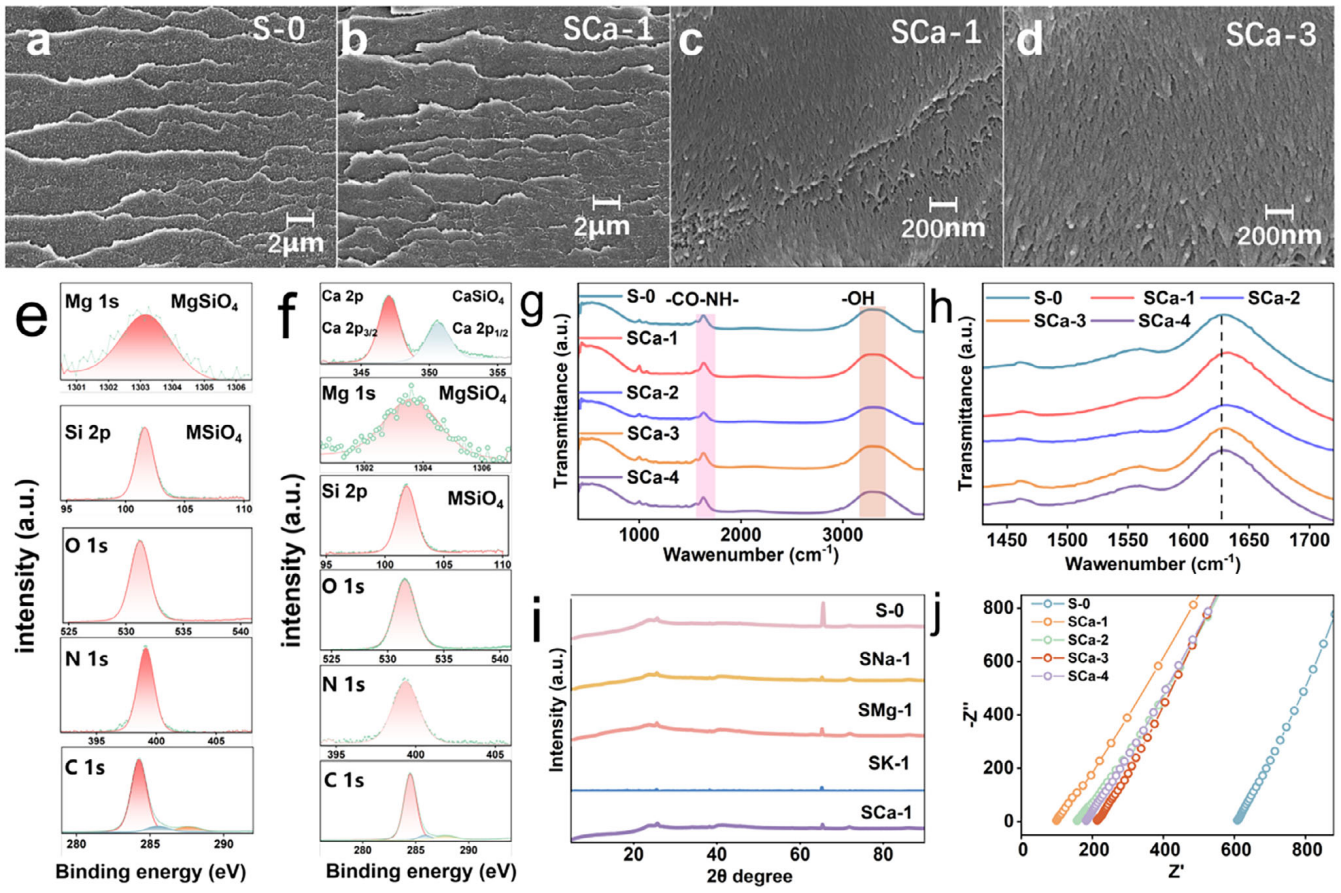
The SEM images of the surface in (a) S‐0 and (b)SCa‐1. (c) The high‐resolution SEM images of the cross‐section in (c) SCa‐1 and (d) SCa‐3. XPS spectra of (e) S‐0 and (f) SCa‐1. (g,h) FTIR spectra of gels. (i) XRD tests of gels. (j) Electrochemical impedance spectroscopy of gels.

Scanning electron microscopy was performed on dried samples, revealing that S‐0 exhibits a distinct two‐dimensional layered surface structure (Figure 2a) with hierarchical interfaces [39, 40]. As shown in Figure 2b and Figure S1), which demonstrates that the incorporation of metal ions preserves a robust layer‐stacked architecture [39]. This structure not only maintains its original thermochromic characteristics but also enhances the shielding performance of the gel. High‐resolution observation of the SCa‐1 and SCa‐3 cross‐section (Figure 2c,d) reveals densely packed striations arranged in an ordered lattice‐like pattern, providing strong evidence for the layer‐stacked configuration. XPS spectra were employed to characterize the material composition through comparative analysis of samples S‐0 and SCa‐1. As depicted in Figure 2e,f, the calibrated main peak of carbon (C) is located at 284.8 eV. SCa‐1 exhibits the calcium elemental spectrum with two characteristic peaks at 348 and 352 eV. These peaks are attributed to the 2p orbital splitting of Ca^2^^+^, corresponding to the 2p_1_/_2_ and 2p_3_/_2_ spin–orbit components, respectively [40]. The silicon element, predominantly present in the form of silicate structures within the system, exhibits a corresponding characteristic peak at 104 eV. FT‐IR spectroscopy was employed to characterize the chemical interaction of gels. As illustrated in Figure 2g, the characteristic peak in the 3000–3500 cm^−^^1^ range confirms the abundance of hydroxyl groups within the system [41]. The distinct peak at 1300 cm^−^^1^ corresponds to the presence of amide groups. Notably, the formation of coordination bonds between calcium ions and amide groups leads to reduced tensile vibration intensity of the amide moieties, accompanied by a slight redshift of this characteristic peak, as displayed in Figure 2h [42]. To characterize the lattice features of the layered structure, XRD tests were performed as depicted in Figure 2i. S‐0 possesses the strongest lattice characteristic peak that appears at 65°, corresponding to the crystalline structure of Laponite XLG [43]. The incorporation of calcium ions caused a significant reduction in the intensity of this characteristic peak. This phenomenon arises from the ion exchange, which disrupts the crystalline order and consequently diminishes the diffraction peak intensity. This result indicates that many exchanged ions are generated within the system [44], which is reflected in the electrochemical impedance characteristics of the material. S‐0 exhibits the highest characteristic impedance, while the impedance of SCa gels shows a gradual decreasing trend (Figure 2j. This phenomenon is attributed to the generation of free ions after ionic coupling in the gels, which can act as charge carriers to migrate in the electric field and conduct as a local current. When excited by electromagnetic waves, these ions migrate in the alternating electric field [45], converting the energy of the electromagnetic waves into thermal energy loss.

### 2.2 Electromagnetic Shielding Characteristics of Layered Gels throughout the Entire Process

**FIGURE 3:**
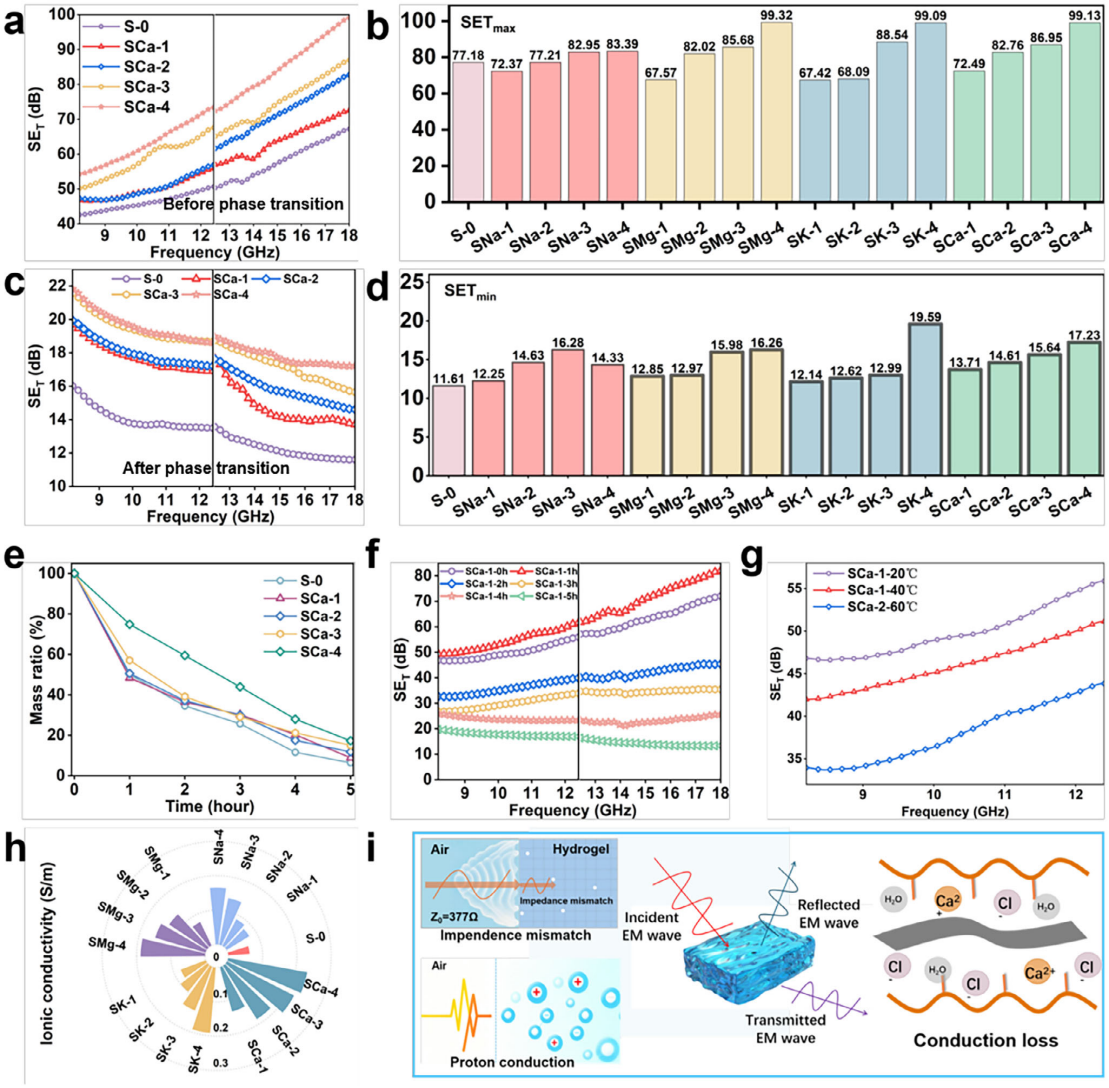
(a) Electromagnetic shielding effectiveness of SCa gels before phase transition. (b) The change pattern of the maximum electromagnetic shielding efficiency. (c) Electromagnetic shielding effectiveness of SCa gels after 5 h of phase transition at 60°C. (d) The change pattern of the minimum electromagnetic shielding efficiency after 5 h of operation at 60°C. (e) Mass ratio at different working times. (f) The variation in electromagnetic shielding performance of SCa‐1 throughout the entire process. (g) Electromagnetic shielding effectiveness of SCa‐1 at different temperatures. (h) Ionic conductivity of gel. (i) Electromagnetic shielding mechanism in gel.

The electromagnetic shielding performance of the materials is illustrated in Figure 3a and Figures S2 and S3. SCa‐1 exhibits a maximum electromagnetic shielding effectiveness (SE) of 72.49 dB, which can even shield over 99.9999% of EM waves, providing effective electromagnetic protection in both the X‐band and Ku‐band [46]. The metal ion‐coupled gels all demonstrate enhanced electromagnetic shielding capabilities; the higher the ion addition, the better the shielding performance (Figure 3b). This enhancement is primarily attributed to the enhanced conductivity of the material, which promotes the reflection and energy attenuation of electromagnetic waves [47]. Ca^2+^ and Mg^2+^ primarily cross‐link with the network through covalent interactions. Their coordination with Nipam generates additional cross‐linking sites that interweave with Laponite XLG to form a more robust layered structure. This framework stabilizes water molecule retention within the gel, thereby enhancing its proton conduction [48]. In comparison, Ca^2+^ has a lower charge density; the network structure it forms upon binding with the gel is more loose, resulting in greater swelling and water‐retention capacity. This allows it to accommodate more water molecules, thereby providing superior shielding efficiency. Additionally, a few metal ions exchange with Na^+^ in Laponite XLG to increase the concentration of free charge carriers, which augments electromagnetic energy dissipation. In contrast, Na^+^ and K^+^ primarily exist in ionic form, which enhances the charge migration to boost the conductive capacity of gels. Therefore, these metal ions all possess the ability to enhance the conductivity of the gel, increasing the electromagnetic shielding effectiveness of the composite.

Considering the impact of the thermochromic properties on its shielding performance, a 5 h simulation at 60°C was conducted to evaluate its performance under extreme conditions. The quality of the gel will hardly change again. The phase transformation causes the loss of abundant water molecules during this period. The electromagnetic shielding effectiveness of the material at this point is shown in Figure 3d; Figures S4 and S5). The S‐0 exhibits the lowest shielding effectiveness at 11.61 dB. In contrast, the gels coupled with metal ions demonstrate enhanced shielding effectiveness. The performance of metal ion‐coupled gels still increases with the rising addition of metal ions, showing a similar change pattern with that of the Initial state (Figure 3d). These results confirm that the incorporation of metal ions enables a marked improvement in electromagnetic protection capability while preserving the inherent structural merits of the material.

Monitoring the entire phase transition process, as shown in Figure 3e, reveals a gradient decrease in gel mass as water content diminishes. To confirm that the material can achieve full process electromagnetic shielding, we conducted a comparative analysis of the shielding effectiveness between S‐0 and SCa‐1, as depicted in Figure 3f and Figure S6. It can be observed that as operating time increases, the shielding effectiveness of the materials gradually decreases. The shielding effectiveness of the optimized SCa‐1 sample remained higher than S‐0, showing a minimum shielding effectiveness of 13.71 dB. This result demonstrates that the gel can sustain continuous operation at 60°C while maintaining highly effective shielding against electromagnetic radiation throughout the entire process. Due to its consistently higher absorption efficiency than reflection efficiency, the material is recognized as an absorbing electromagnetic shielding material (Figure S7).

As shown in Figure 3g, the shielding effectiveness of the material decreases with the temperature increase, which is attributed to the slight decrease in the complex dielectric constant and loss tangent angle of the material caused by the loss of water, resulting in weaker absorption efficiency of the material (Figure S8). This phenomenon proves that the performance changes of the material are closely related to the water molecules in the system. Additionally, the conductivity of materials is a favorable evidence to prove the optimization effect of metal ions, which primarily stems from ion migration. Since no electronically conductive materials exist within the gel, the conductivity of materials can be approximated by ionic conduction. Their ionic conductivity, as shown in Figure 3h, increases steadily with rising metal ion concentration. This indicates that more metal ions acting as charge carriers enhance the conduction current, dissipating electromagnetic wave energy as Joule heat in the conduction process [49]. The electromagnetic shielding mechanism of the material is summarized as shown in Figure 3i. The reflection of electromagnetic waves is caused by impedance mismatch due to the differences in conductivity and dielectric properties between air and the material [50]. The absorption of electromagnetic waves primarily occurs through conduction loss, which results from the synergistic effect of proton conduction and the migration of metal ions.

**FIGURE 4:**
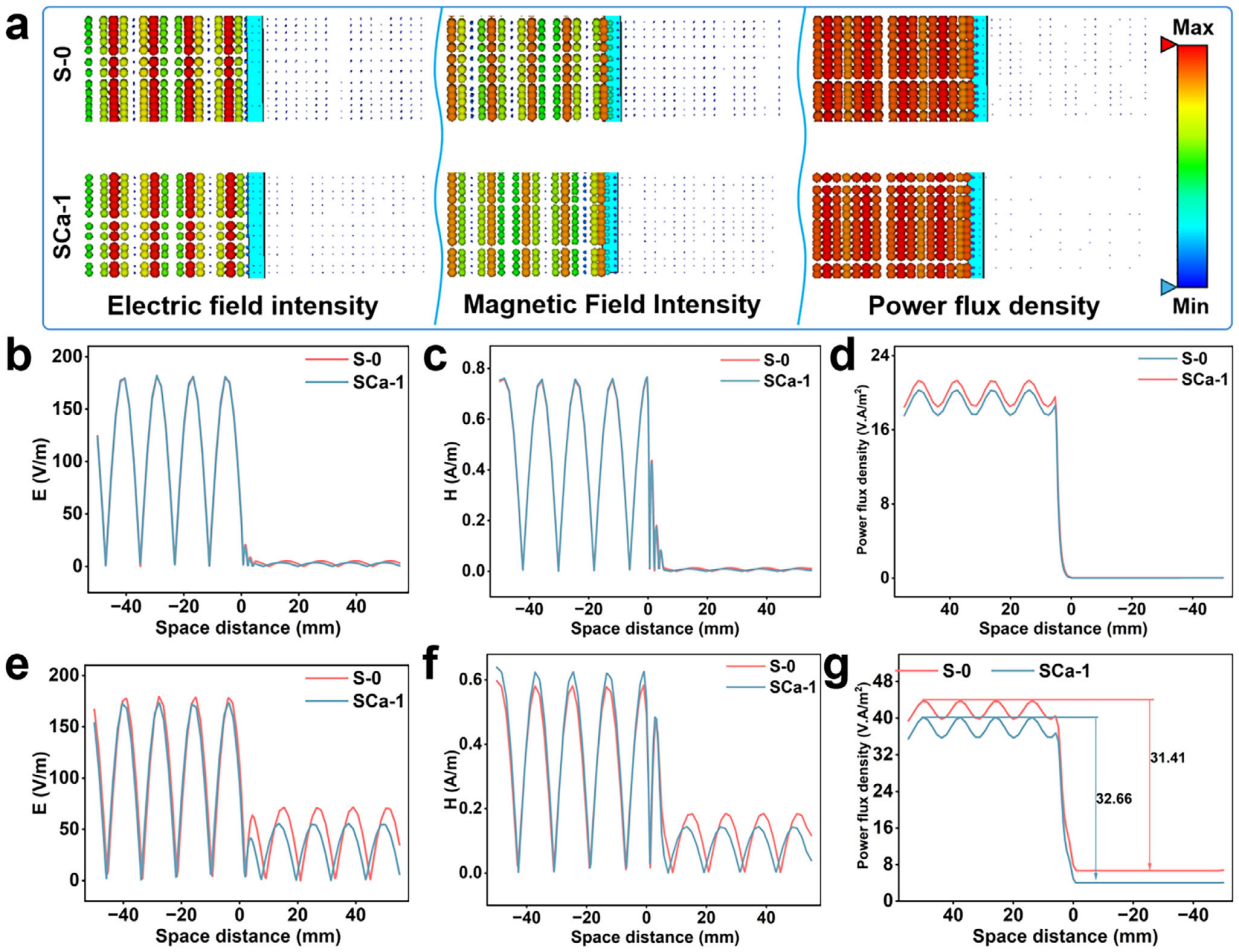
(a) CST simulation of electromagnetic field intensity and power flux density in space for S‐0 and SCa‐1 at the initial state. Distribution curve of (b) electric field, (c) magnetic field, and (d) power density of the initial gel in space for gels at the initial state. Distribution curve of (e) electric field, (f) magnetic field, and (g) power flow density of initial gel in space for gels after 5 h operation at 60°C.

The actual shielding effect of the material can be verified via the actual shielding performance of the material can be verified via CST simulations. In the standard antenna array space, the intensity of the excited electromagnetic field and the power flux density are presented in Figure 4a. The transmitted electromagnetic waves induce an extremely weak electromagnetic field, with a significant reduction in power flux density. Specifically, SCa‐1 exhibits superior shielding performance, achieving nearly complete shielding of electromagnetic waves. The spatial electric field monitoring results are in Figure 4b,c, the incident electromagnetic wave with an excitation electric field amplitude of 180 V/m transmitted through the material, the amplitude decreases to 5 V/m, while the excitation magnetic field decays to nearly 0. The power flux density distribution across the entire space further confirms that almost all electromagnetic wave energy is blocked outside the material (Figure 4d). As shown in Figure S9, the material still maintains effective electromagnetic protection capability after operating at 60°C for 5 h. The specific electric field distribution is displayed in Figure 4e,f. The amplitude of the electric field excited by SCa‐1 decreases from 175 to 55 V/m, and the magnetic field intensity is reduced by approximately 77%. Additionally, the power flux density distribution diagram demonstrates that the material can shield electromagnetic radiation with high efficiency (Figure 4g).

### 2.3 Thermochromic Properties of Layered Gels

**FIGURE 5:**
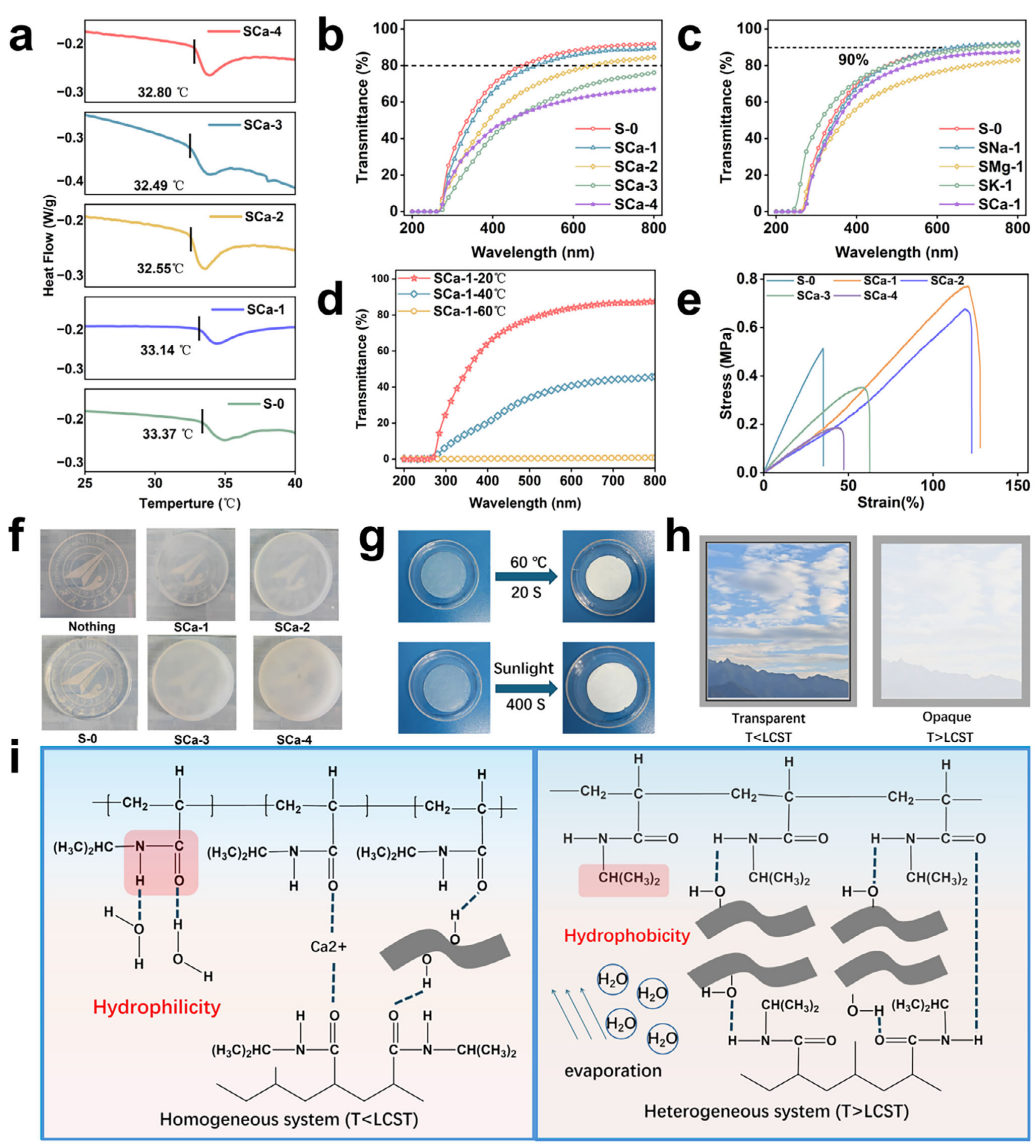
(a) DSC tests of gels. (b,c) Transmittance spectra of gels. (d) Transmittance spectra of gels at different temperatures. (e) Tensile properties of gels. (f) The Photograph of SCa gels. (g) Thermochromic properties of SCa‐1 at different temperatures. (h) schematic diagram of a smart window. (i) Thermochromic mechanism of the gel system.

The phase transition characteristics of the material were characterized by DSC. As shown in Figure 5a, S‐0 exhibits the highest phase transition temperature. The addition of calcium ions promotes a decrease in this phase transition temperature [51]. When calcium ions bind to amide groups as strong hydrophiles, whose affinity for water is stronger than that of Nipam. This reduces the attraction of Nipam chains to water molecules, promoting hydrophobic interactions and consequently reducing the phase transition temperature. Conversely, the increase in phase transition temperature for SCa‐4 is attributed to excessively high calcium ion concentration, which absorbs more water, resulting in a slight elevation of the required transition temperature. To evaluate the optical properties of the gels, their transmission spectra are presented in Figure 5b,c. The transmittance of the SCa gel decreases with increasing calcium ion content, as the formation of coordination bonds introduces additional cross‐linking points. This leads to a heterogeneous network structure and consequent reduction in light transmission. This phenomenon is less pronounced in SNa‐1 and SK‐1, which rely on noncovalent cross‐linking, which indicate that metal ions exert a limited regulatory effect on the network structure through non‐covalent interactions. SCa‐1 exhibits a high visible light transmittance (*T*_lum_) of 83.49% and a solar transmittance (*T*_sol_) of 83.20%, with Δ*T*_lum_ and ΔT_sol_ reaching 82.57% and 83.05% (Figure S10), respectively. These results confirm its excellent light transmission capability and high energy efficiency. Transmittance spectrum measurements of SCa‐1 at different temperatures (Figure 5d) reveal that at 60°C, following the phase transition, the material becomes nearly opaque. These findings confirm the excellent thermochromic performance of materials, enabling dynamic regulation of their optical properties and achieving synergistic compatibility with electromagnetic shielding applications. The mechanical properties of gels reflect their durability and compressive strength. The S‐0 sample exhibits the poorest ductility at only 35%. As calcium ion content increases, SCa‐1 achieves a maximum tensile elongation of 127%, which is about 3.62 times higher than S‐0 (Figure 5e). The improvement stems from the covalent interactions between calcium ions and hydroxyl. Under external force excitation, hydrogen bonds first break as sacrificial bonds. Covalent bonds keep their original state due to their high bond energy, ensuring excellent stability while maintaining their layer structure. However, the mechanical properties of SCa‐3 and SCa‐4 deteriorate due to excessive calcium ions, causing a sharp increase in cross‐link density within the system. This eliminates deformation of buffer space in the gel network, leading to brittle fracture when external force reaches a critical threshold.

The schematic diagram of gels demonstrates exceptional optical transmittance, rendering it highly suitable for smart window applications with superior visual clarity, as shown in Figure 5f and Figure S11. We conducted temperature testing with SCa‐1. At 60°C, the material will quickly transition to a white opaque state after 20 s. In weather with a temperature of 40°C, the sample will also transform into an opaque state after 400 s of response (Figure 5g). This result indicates that the material can respond quickly in environments above its LCST, thereby achieving intelligent temperature control of visible light absorbance. Leveraging these unique thermochromic characteristics, the material can function as a smart window. As illustrated in Figure 5h, when the temperature exceeds its LCST, the gel transitions from a transparent to an opaque state, significantly blocking visible light from outside [52].

This unique phase transition mechanism is shown in Figure 5i. When the temperature of the system is below the LCST, the amide groups in the Nipam molecular chains dominate the hydrophobic interaction. This occurs because the thermal motion of water molecules is relatively weak at this temperature, resulting in an ordered molecular arrangement. The functional groups on the Nipam chains interact with water molecules via hydrogen bonds, exhibiting pronounced hydrophilic behavior. Laponite XLG also forms an ordered layered structure and stably associates with Nipam, creating a homogeneous gel network. The entire material constitutes a homogeneous system. When light passes through the gel, the uniform refractive index across all regions prevents significant refraction, reflection, or scattering. Instead, light transmits directly, resulting in a transparent state. However, when the temperature exceeds the LCST, the isopropyl groups in the Nipam molecular chains dominate. This shift occurs because the thermal motion of water molecules intensifies dramatically, generating sufficient energy to break the hydrophilic hydrogen bonds between Nipam and water molecules. Concurrently, the hydrophobic interactions of the isopropyl groups are significantly enhanced by entropy increase, ultimately manifesting as the hydrophobic properties of the gel. During this process, the amide group weakens the interaction with water molecules, degrading from strong hydrogen bonding to weak adsorption. The decrease in the hydrophilic capacity of the amide groups leads to the breakage of hydrogen bonds, thereby causing the water molecules originally enveloping the segments to be expelled. The segments lose the protection of the hydration layer, and the nonpolar isopropyl groups approach each other, triggering hydrophobic aggregation of the PNipam segments. Simultaneously, the contraction of the three‐dimensional cross‐linked network gives rise to microscopic phase separation. It results in a microphase structure comprising a polymer‐rich phase and a water‐rich phase, rendering the entire system distinctly heterogeneous. This heterogeneity makes light to undergo multiple refractions, reflections, and scatterings at the phase interfaces, manifesting macroscopically as an opaque state [53, 54].

**FIGURE 6:**
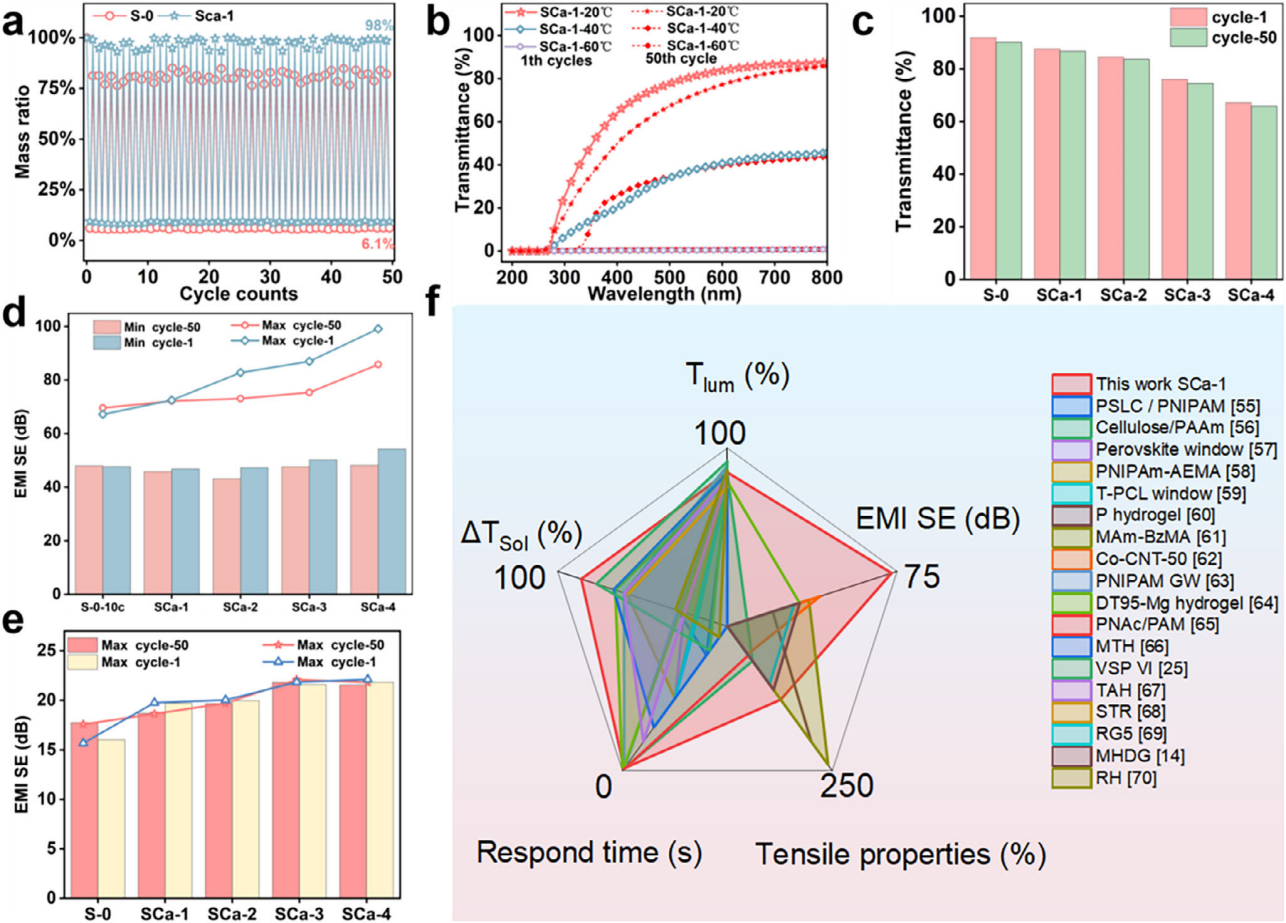
(a) Mass ratio at fifty recycling. (b) Thermochromic properties of SCa‐1 at the initial state and the fifty cycles. (c) Comparison of the maximum transmittance between the initial state and the fifty cycle. Comparison of electromagnetic shielding effectiveness (d) before phase transition and (e) after phase transition. (f) Comprehensive Performance Comparison.

To demonstrate the practicality, functionality, and environmental adaptability of the materials, we conducted cyclic application tests. As shown in Figure 6a and Figure S12, S‐0 exhibits significant mass loss after fifty cycles due to its weak internal network association, which results in inferior water retention and absorption capabilities, preventing timely recovery to the working state. When calcium ions are introduced into the gel system, they function as cross‐linking agents, reinforcing the gel network and enhancing the interaction with water molecules. This is attributed to calcium ions binding with water molecules in aqueous environments to form hydrated ions, accelerating the adsorption of water molecules and facilitating rapid recovery to the working condition. After fifty operational cycles, the transmission spectra of the material at different temperatures are presented in Figure 6b, with thermochromic properties similar to those observed in the first cycle. This result confirms that the gel retains excellent light‐modulation capabilities over extended periods. Comparing the maximum light transmittance of the samples before and after the cycling test, the optical properties slightly decreased after fifty cycles (Figure 6c; Figure S13). This phenomenon primarily stems from the network restructuring during repeated swelling‐deswelling cycles, which affects the microstructure of the gel and reduces the homogeneity of the system. Considering the electromagnetic shielding performance of materials during multiple cycles of operation (Figure S14), we focused on analyzing the performance changes of the gel after the fifty cycle, as shown in Figure 6d. The shielding effectiveness of SCa‐1 is consistently higher than 44.53 dB, shielding 99% of electromagnetic radiation. This indicates that it can still function normally. Comparing this further with the electromagnetic shielding characteristics during the first operation, significant performance changes are observed in SCa‐2, SCa‐3, and SCa‐4, which are attributed to the excessive gel cross‐linking density, which reduces fatigue resistance and makes it difficult to recover. The electromagnetic shielding effectiveness after complete phase transition, as shown in Figure 6e, after the fifth cycle of operation, the SCa‐1 delivers optimal performance of up to 18.52 dB, with all samples achieving shielding effectiveness exceeding 10 dB (Figure S15). Furthermore, the shielding effectiveness of materials remains virtually unchanged. This confirms that the material maintains reliable electromagnetic shielding throughout service and provides highly efficient protection. Consequently, it fulfills dual functionalities as both an electromagnetic shielding material and a smart window, while enabling continuous and repeated cyclic use.

To comprehensively evaluate the performance as a dual‐functional smart window and electromagnetic shielding material, we synthesized and compared the key properties of multiple candidate materials, as illustrated in Figure 6f [14, 25, 55, 56, 57, 58, 59, 60, 61, 62, 63, 64, 65, 66, 67, 68, 69, 70]. SCa‐1 exhibits both high visible light transmittance (*T*_lum_) and large transmittance modulation range (Δ*T*_lum_), representing a significant advantage over comparable smart window materials. Furthermore, it features an exceptionally rapid response speed, enabling swift regulation of light transmittance. Notably, the electromagnetic interference shielding effectiveness (EMI SE) is superior to gel‐based counterparts, providing efficient protection of electromagnetic radiation. SCa‐1 exhibits an elongation at break of 127%, which is 2.62‐fold higher than that of S‐0, along with a high tensile strength at break of 0.72 MPa. Compared to pristine aqueous ionic poly (NIPAM) gels [71, 72]. This result confirms the enhanced structural stability of materials.

## 3 Conclusion

This study presents a layered, structurally interactive gel network constructed from Laponite XLG and NIPAM, demonstrating that the hierarchical architecture promotes the enhancement of ionic conductivity and heat conduction. The electromagnetic shielding and thermochromism characteristics are effectively modulated through coordination with metal ions. Specifically, Ca^2^^+^ and Mg^2^^+^ ions form covalent interactions with amide groups in the polymer matrix, thereby stabilizing bound water molecules within the system to enhance proton conductivity. Additionally, ion exchange generates additional migration charges. In contrast, Na^+^ and K^+^ are free ions within the gel network, allowing for their migration under electromagnetic excitation and contributing to electromagnetic energy dissipation through conductive current loss. The optimal SCa‐1 sample delivers the highest electromagnetic shielding effectiveness of up to 72.49 dB, capable of shielding over 99.9999% of electromagnetic radiation. This distinctive architecture minimizes transfer resistance, which facilitates thermochromism quickly. It achieves a *T*_lum_ of 86.39% and a Δ*T*_sol_ of 83.05%, offering substantial modulation amplitude. We have experimentally validated the efficient operation as a smart electromagnetic shielding window. elucidating its high electromagnetic shielding effectiveness, which arises from impedance mismatch. The phase transition mechanism of the material has been systematically investigated, providing a comprehensive understanding of the structural and dynamic changes that enable its temperature‐responsive behavior.

## Conflicts of Interest

The authors declare no conflicts of interest.

## Supporting information




**Supporting File**: advs75518‐sup‐0001‐SuppMat.docx.

## Data Availability

The data that support the findings of this study are available from the corresponding author upon reasonable request.
