# Supplementary material for: Self‐Assembly Ordered Layered Gels for Thermochromic Smart Windows With Excellent Electromagnetic Shielding Properties
Source: Adv Sci (Weinh). 2026 May 7;13(41):e75518. doi: 10.1002/advs.75518 (PMC13335671; doi:10.1002/advs.75518)
Supplement: Supplementary file 1 — Supporting File: advs75518‐sup‐0001‐SuppMat.docx. [file ADVS-13-e75518-s001.docx]

**Supporting information**

**Experimental procedure**

**Raw materials**

N-Isopropylacrylamide was obtained from Aladdin, Laponite XLG from Guangzhou Daixun Trading Co., Ltd., N, N'-Methylenebisacrylamide, tetramethyl ethylenediamine, sodium chloride, magnesium chloride, potassium chloride, and calcium chloride were all purchased from Macklin Chemical Reagent Co., Ltd. All chemicals were used as received without any purification, and deionized water (>18.2 MΩ) was used throughout this study.

**Preparation of calcium ion-modified Nipam/Laponite XLG composite gel**

Dissolve 0.6 g of Laponite XLG in 18 ml of deionized water, stir at room temperature for 10 minutes until it becomes clear, then add 2 g of Nipam and 0.03 g of MBA, and stir in the dark for 3 hours. Place the resulting clear solution in an ice bath for ten minutes, and then quickly add 10 microliters of tetramethyl ethylenediamine, 1 ml of 0.01 g/ml calcium chloride solution, and 1 ml of 0.03 g/ml ammonium persulfate solution in order, stir for 20 seconds, and then inject into the mold. Place the precursor in a dark environment with a temperature of 10-20℃ for polymerization for 5 hours. Soak the sample in deionized water to achieve swelling equilibrium and name the obtained sample SCa-1.

**Preparation of the control group**

Only the concentration of added metal ions was changed. Following the same experimental steps and drug dosages, the sample without added metal ions was designated as S-0, and samples prepared with 0.02g/ml, 0.03g/ml, and 0.04g/ml calcium chloride solutions were designated as SCa-2, SCa-3, and SCa-4, respectively. Additionally, SNa-X, SMg-X, and SK-X (X=1, 2, 3, 4) prepared from sodium chloride, magnesium chloride, and potassium chloride solutions were also prepared following the same salt concentration gradient.

**Characterization**

To investigate the optimization effect of metal ions on the electromagnetic shielding effectiveness and thermochromic properties of gels, Fourier-transform infrared spectroscopy (FT-IR), X-ray Photoelectron Spectroscopy (Thermo Kalpha), and X-Ray Diffraction (Ultima IV) are employed to characterize the chemical structure of materials. The layered structure is observed via scanning electron microscopy (SEM, ZEISS Sigma 300, Germany). The transmittance spectra of the gels were measured using an ultraviolet-visible-near-infrared spectrophotometer (Lambda 950, PerkinElmer). An electrochemical workstation (CHI660E) was applied to measure the electrochemical impedance spectrum of the gels. Testing the mechanical properties through the universal electronic testing machine. Measure its phase transition temperature through DSC tests. Vector network (MS46322B) analyzer is used to evaluate the electromagnetic shielding properties of materials and calculate their absorption performance according to transmission line theory. Electromagnetic measurements, ion conductivity and CST simulation are detailed illustrated.

**Cycle tests**

After five hours of heat treatment at 60℃ in a constant-temperature drying oven, simulating extreme weather conditions, the sample quality obtained will remain unchanged, and the changes in quality before and after will be recorded. Subsequently, the completed sample is soaked in a salt ion solution of corresponding concentration, and after one night of swelling equilibrium, it can be restored. Repeat the above experimental procedure and compare the thermochromic performance and electromagnetic shielding effectiveness of the initial sample and the sample that has undergone ten treatments.

**Electrochemical analysis**

The ionic conductivity of the gels was determined using an electrochemical workstation system (CHI660E). Specimens are placed between two polished stainless steel, with a voltage amplitude set to 10mV. Measurements were conducted within a frequency range of 0.01 Hz to 100 kHz. The ionic resistance was obtained by extrapolating the Nyquist curve from the horizontal axis, and the ionic conductivity was calculated using the following formula.

$\sigma_{i}=\frac{L}{A\times R}$ (S1)

where σ_i_ is the ionic conductivity, L is the distance obtained between the two electrode plates, A is the contact area, and R is the ionic resistance.

**CST simulation**

CST Simulation Conditions: The simulation of electric fields, magnetic fields, and power loss density is based on the antenna models in the Microwave and RF/Optics modules of CST, using the waveguide workflow to perform time-domain solutions. The material model is a 100 × 100 × 5 mm rectangular prism. An alternating electromagnetic field with a frequency range of 8.2–12.4 GHz is applied within a 500 mm space above and below the material. An excitation port (Port 1) and a receiving port (Port 2) are defined at the spatial boundaries, with the initial field strength at the excitation port set to 1000 V/m. while the remaining boundaries perpendicular to the electric and magnetic fields are set to E=0 and H=0, respectively. The material’s permittivity and permeability are derived from actual measurements taken with a vector network analyzer. Finally, using the one-dimensional curve tool, the center of the space was selected to calculate the electric field distribution, magnetic field distribution, and power loss density values along the direction of electromagnetic field propagation through the material.

**Electromagnetic shielding effectiveness**

To eliminate the influence of thickness on the material, we used a standardized custom mold to prepare samples with a uniform thickness of 3 mm. This approach was adopted for both the evaluation of the electromagnetic shielding performance and subsequent testing of materials. The parameters of the prepared samples were measured using the waveguide method with a vector network analyzer (MS46322B) over the frequency ranges of 8.2-18 GHz. During the measurements, the gels were carefully sheared into rectangles of specific dimensions to prevent leakage of EM waves. The S-parameters (S11, S21 or S12, S22) are directly measured using the network. In addition, temperature variation testing mainly relies on external heating modules, which are tested after being held at a fixed temperature for 1 minute. The total EMI SE (SE_T_) and contributions from reflection (SE_R_) and absorption (SE_A_) were calculated as follows:

R = |S11| ^2^ = |S22| ^2^ , T = |S21| ^2^ = |S12| ^2^ (S2)

A = 1 − R − T (S3)

SER = −10 log(1 − R), SEA = −10 log(T/(1 − R)) (S4)

SET = SEA + SER (S5)

**Optical performance**

The solar spectrum (200 nm to 2500 nm) was measured using a UV-Vis-NIR spectrophotometer (Lambda 950, PerkinElmer) equipped with a 150 mm integrating sphere. The spectrophotometer was fitted with a heating and cooling stage to regulate sample temperature. Solar transmittance (T_sol_) was calculated according to the following formula:

$T_{\text{sol}}=\frac{\int_{300nm}^{2500nm} T(\lambda)\mathrm{E}_{\mathrm{sol}}(\lambda)\text{d}\lambda}{\int_{300nm}^{2500nm} \mathrm{E}_{\mathrm{sol}}(\lambda)\text{d}\lambda}$ (S14)

Where T(λ) denotes the measured spectral transmittance, and E_sol_(λ) represents the spectral solar radiation power (AM1.5G) at 300 K. The luminance transmittance (T_lum_) is calculated as follows:

 (S15)

Where φ(λ) denotes the standard luminous efficacy function for visual light at wavelengths between 380 and 780 nm, and T(λ) corresponds to the same as above.

Light modulation capacity and solar radiation modulation are evaluated using ΔTlum and ΔTsol, respectively, with the calculation formulas as follows:

$\Delta T_{\text{sol}}=T_{\mathrm{sol},20\text{℃}}-T_{\mathrm{sol},60\text{℃}}$ (S16)

$\Delta T_{\text{lum}}=T_{lum,20\text{℃}}-T_{lum,60\text{℃}}$ (S17)


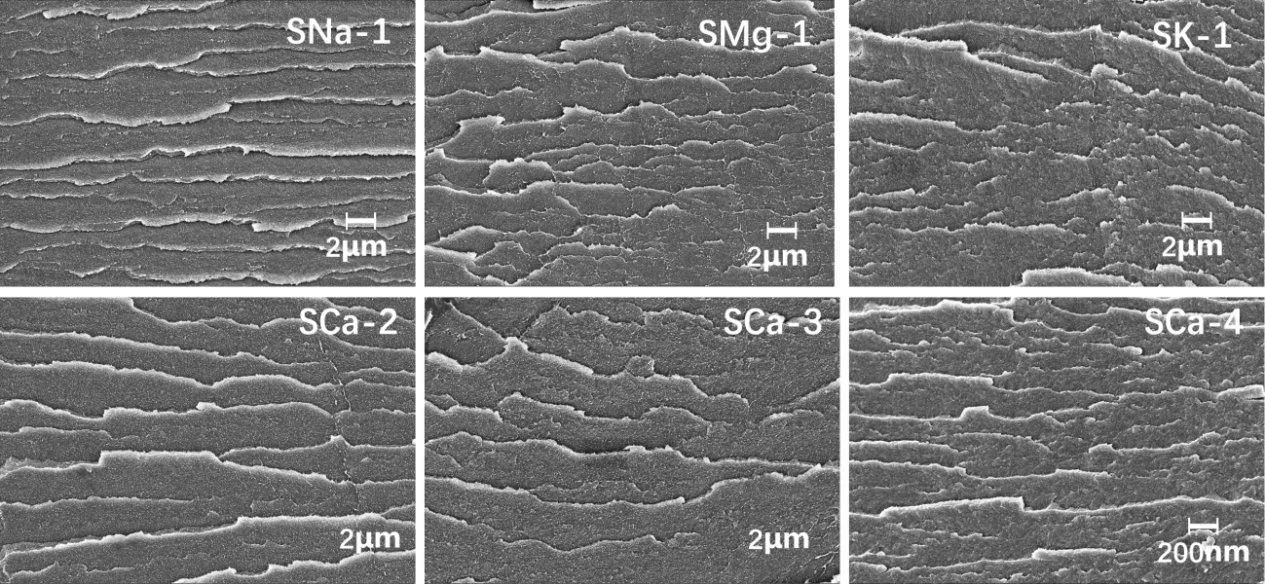


S1. The SEM images of the surface


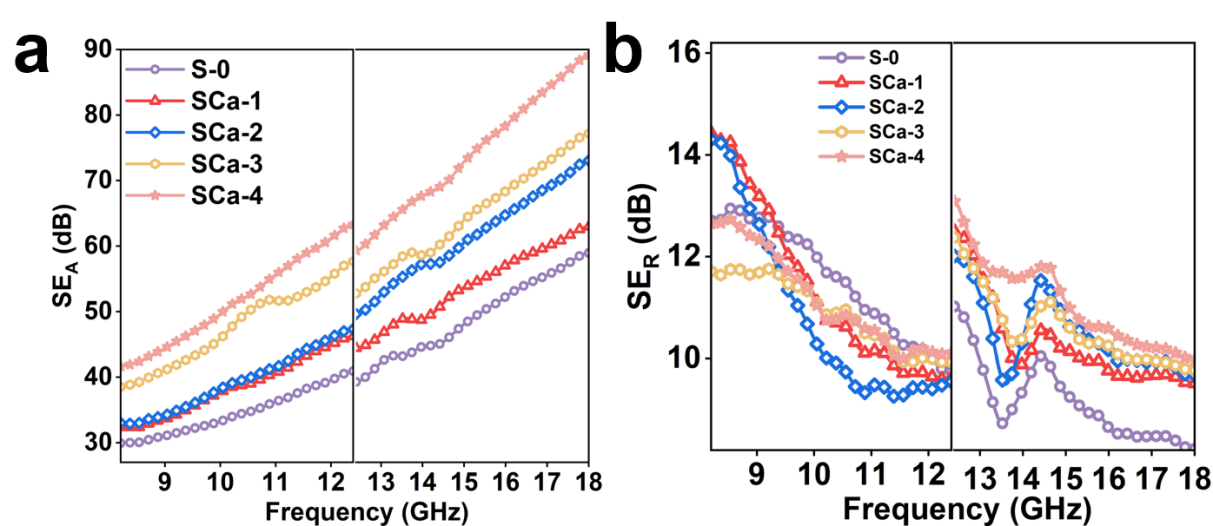


S2. (a) SE_A_ and (b) ST_R_ of SCa gels


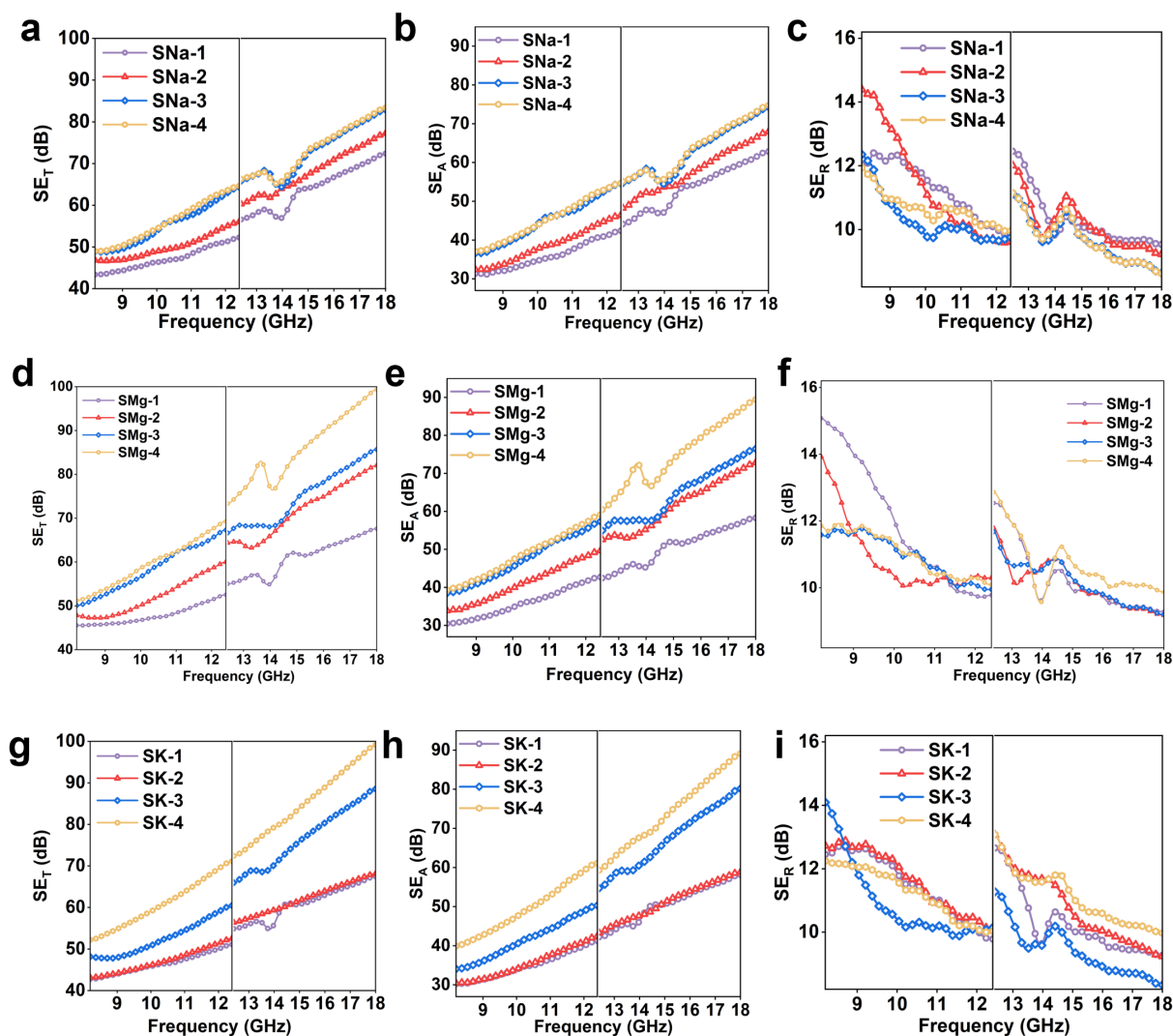


S3. Electromagnetic shielding performance of SNa, SMg, and SK gels


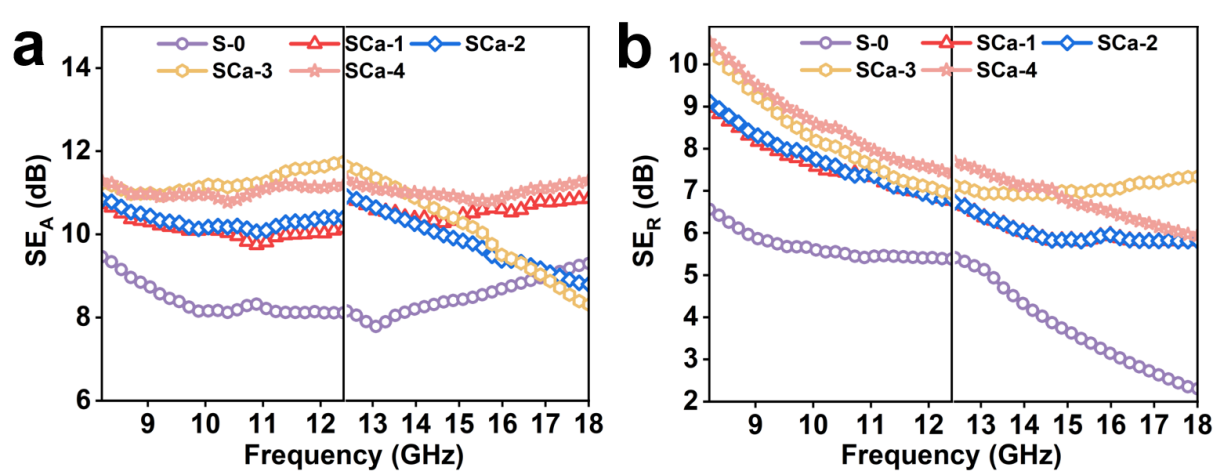


S4. (a) SE_A_ and (b) ST_R_ of SCa gels after 5 hours of operation.


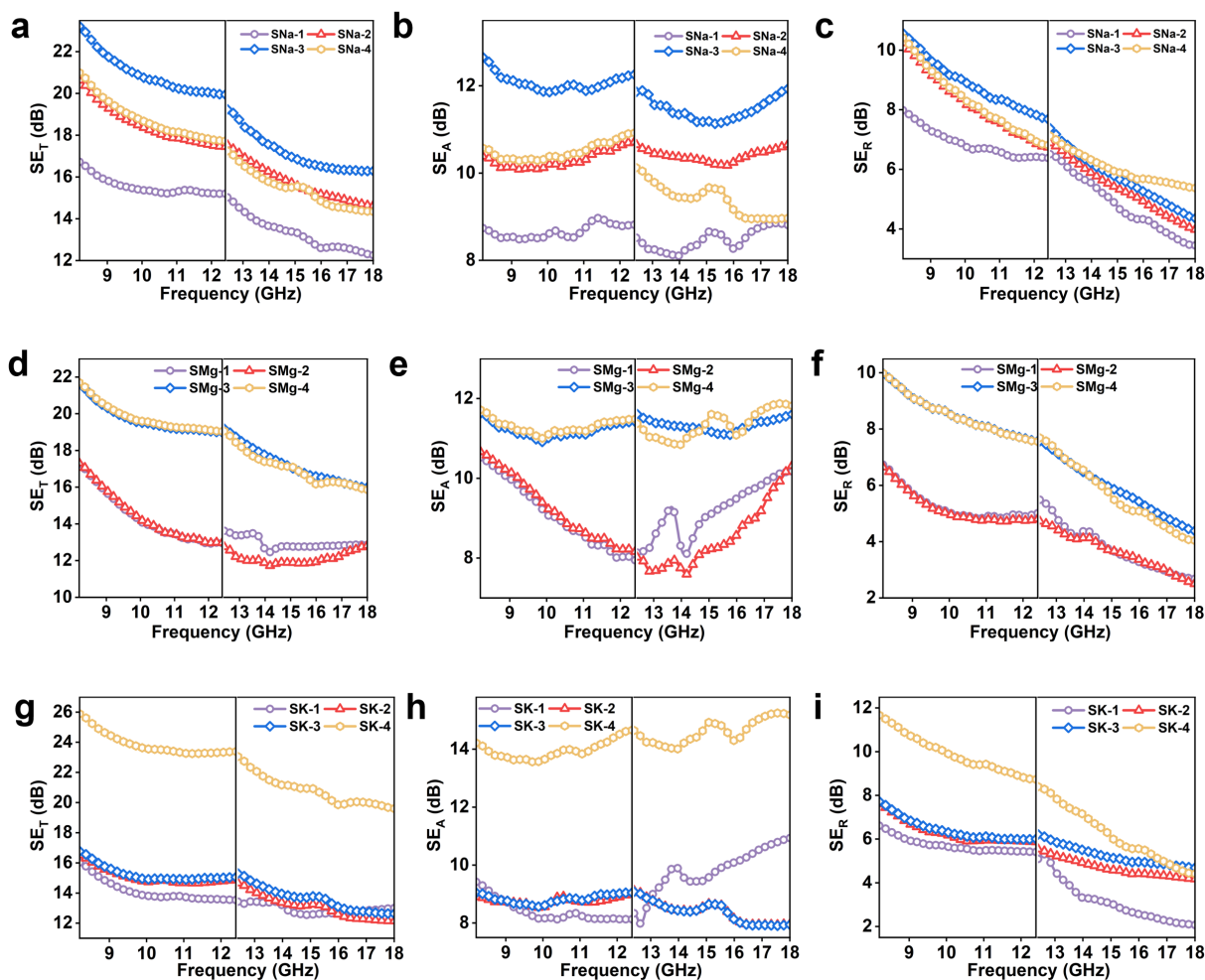


S5. Electromagnetic shielding performance of SNa, SMg, and SK gels after 5 hours of operation.


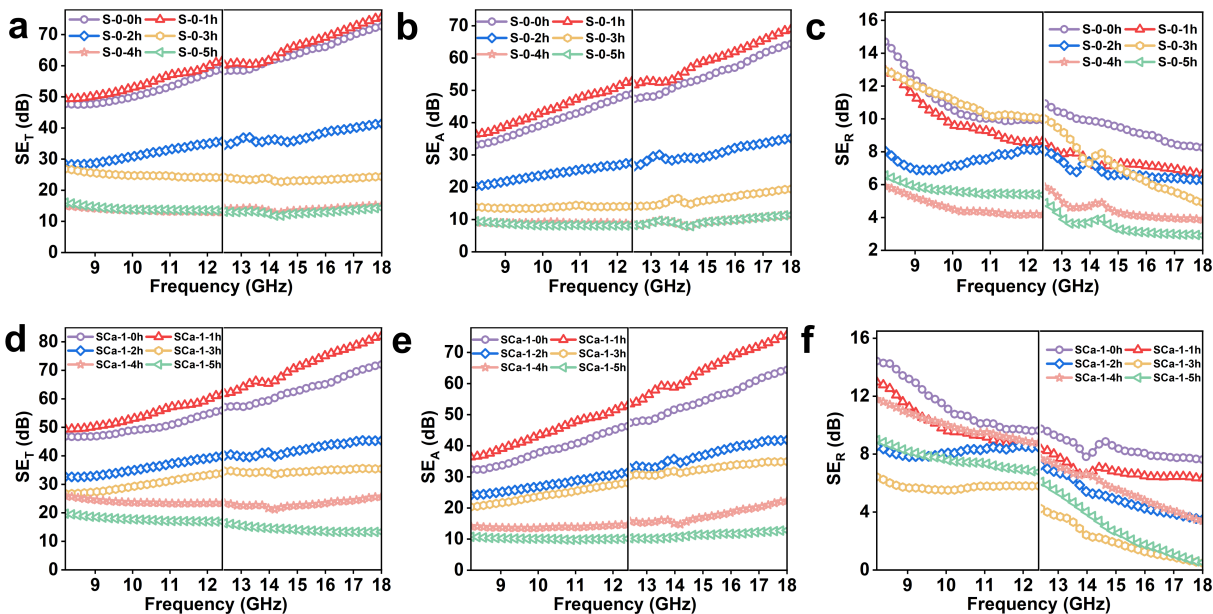


S6. Electromagnetic shielding performance of S-0 and SCa-1 at different work time.


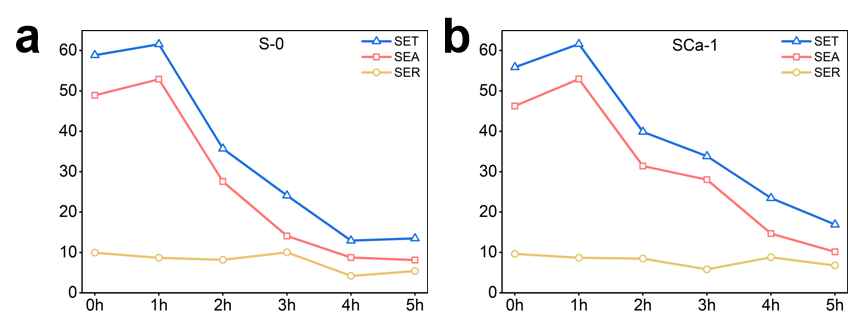


S7. SET, SEA, and SER values for S-0 (a) and SCa-1 (b) at 12.4 GHz


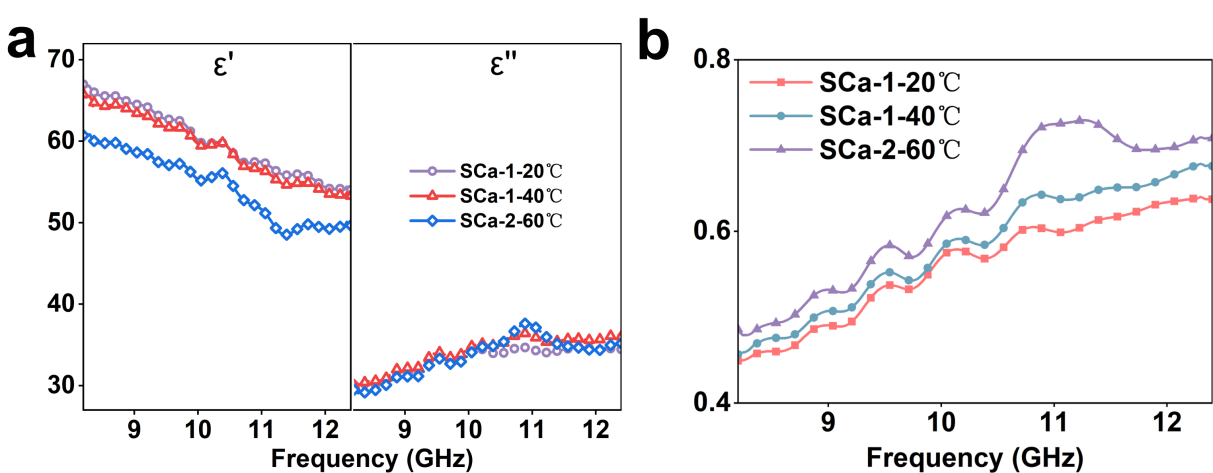


S8. (a) Complex permittivity and (b) Loss tangent angle of SCa-1 at different temperatures.


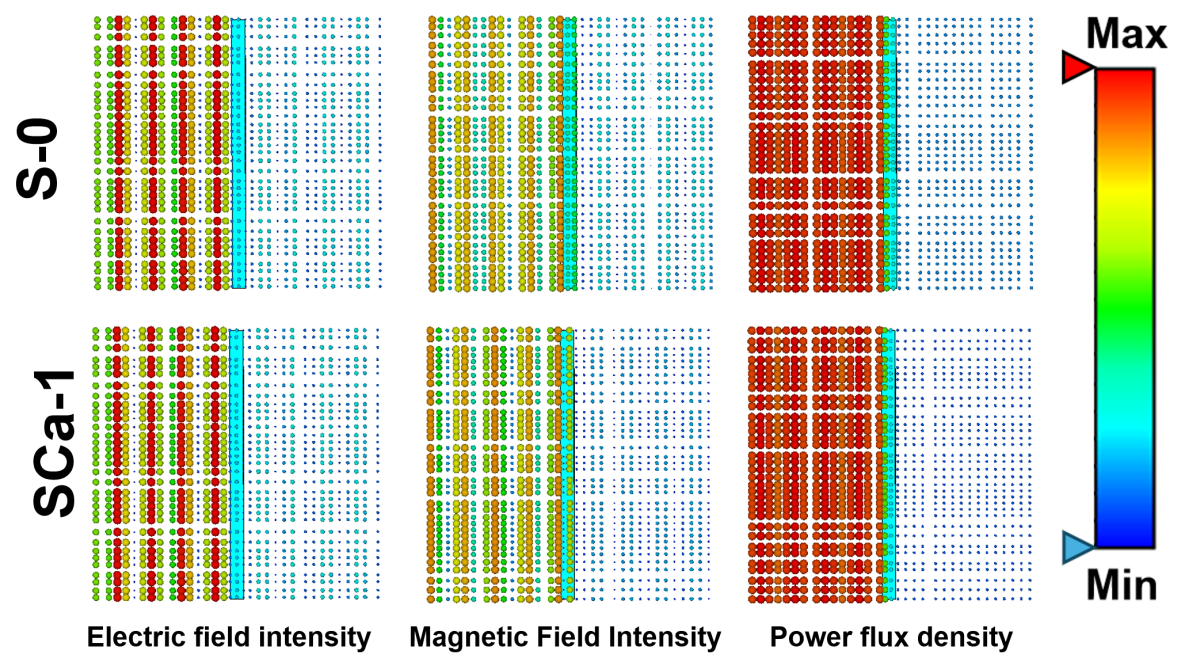


S9.CST simulation of electromagnetic field intensity and power flux density in space for S-0 and SCa-1 after 5 hours operation at 60℃.


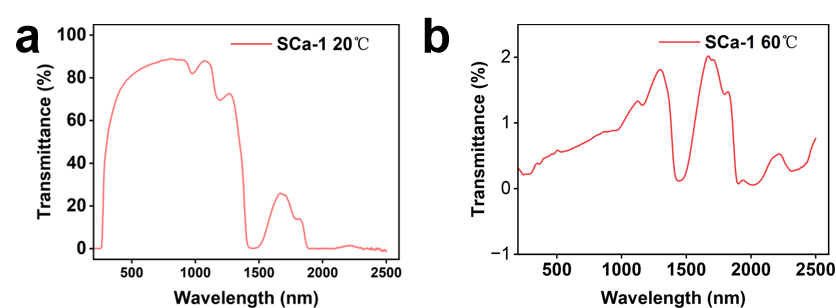


S10. Full-spectrum solar transmittance of SCa-1 at 20°C (a) and 60°C (b)


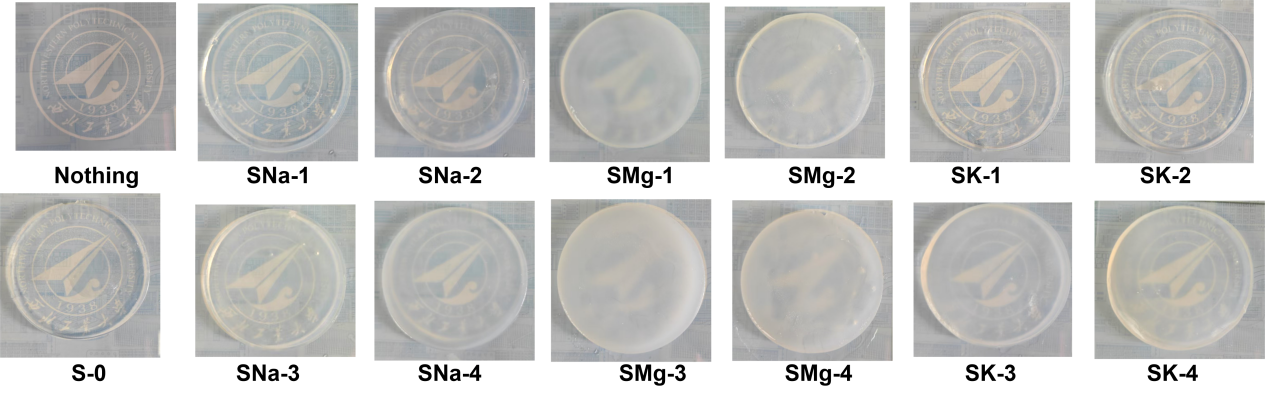


S11. The Photograph of gels


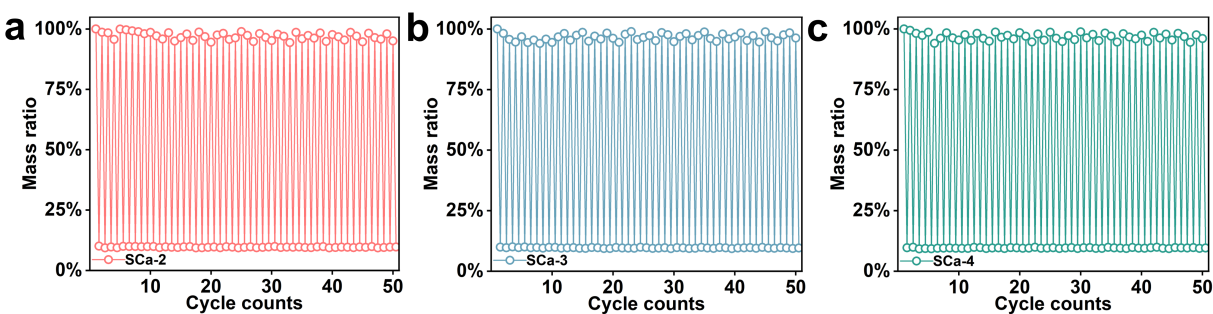


S12. Mass ratio at multiple recycling


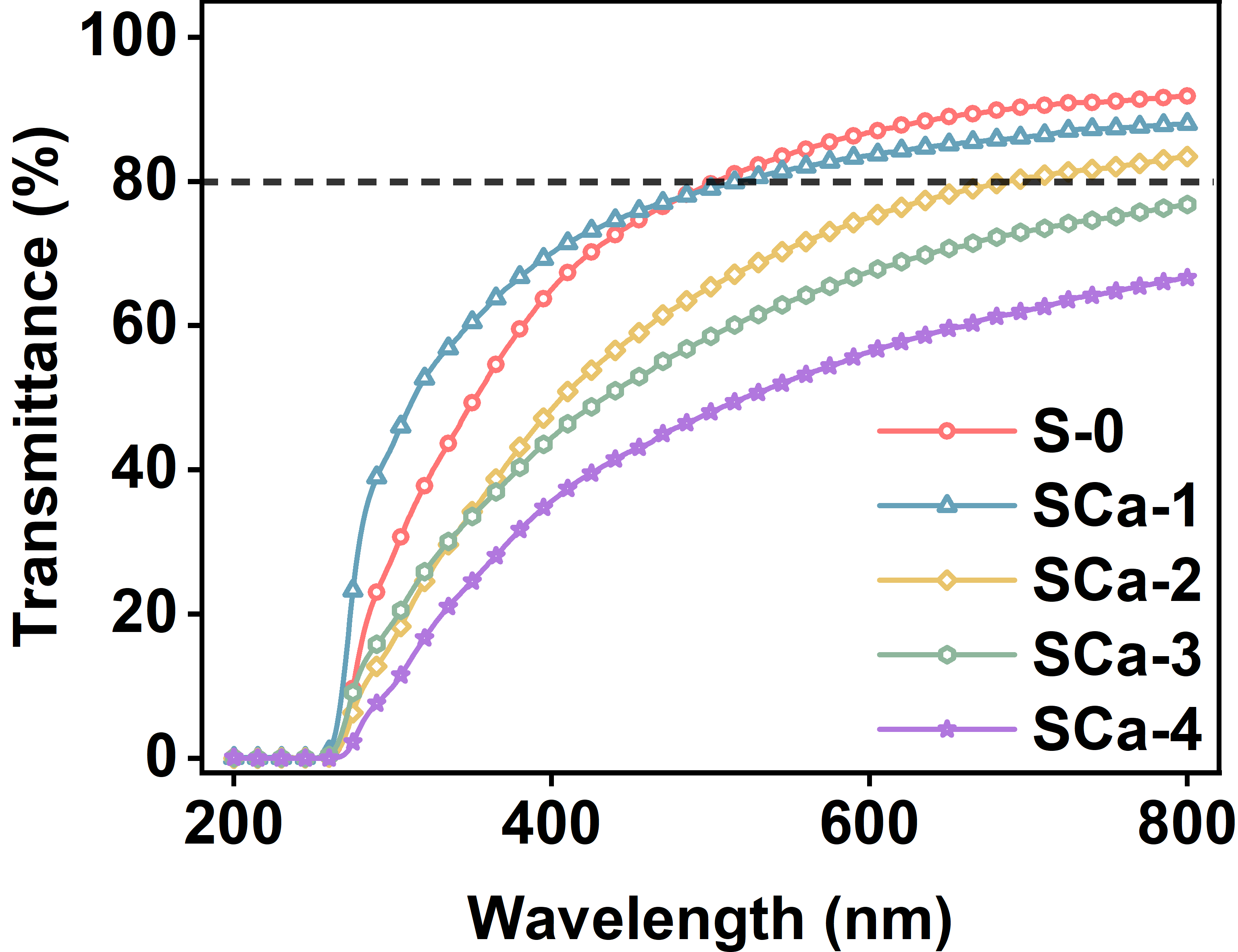


S13.Light transmittance of the sample after 50 cycles


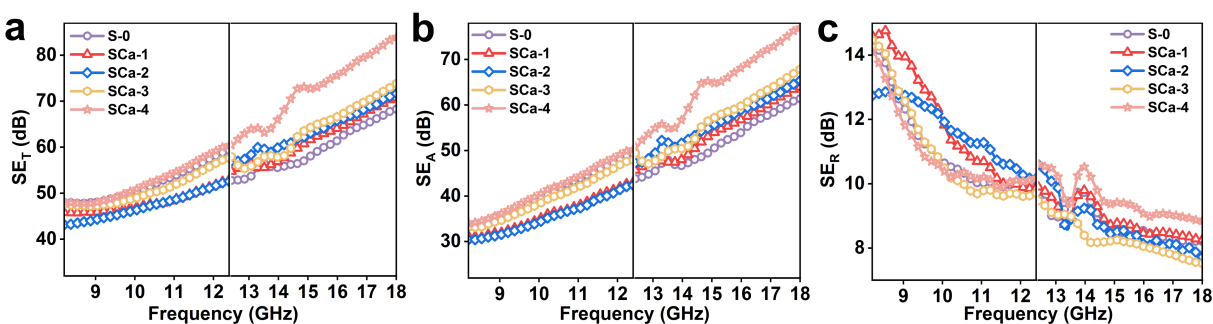


S14. Electromagnetic shielding performance of SCa gels after the 50 cycle.


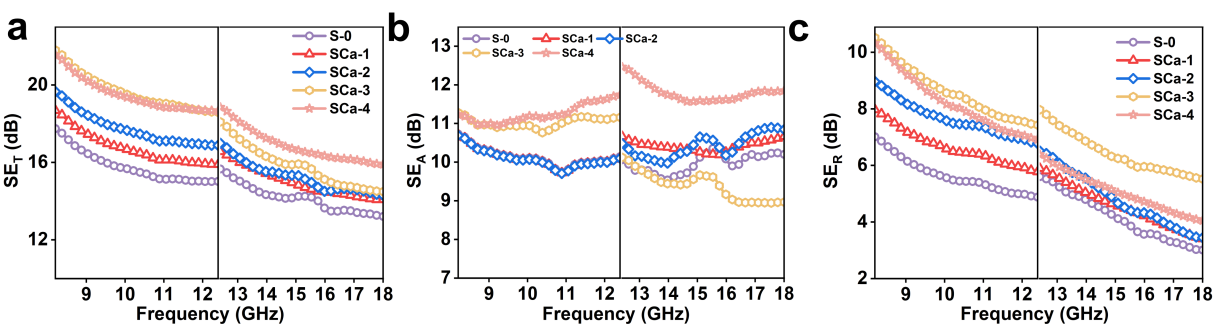


S15. Electromagnetic shielding performance of SCa gels after phase transition after the 50 cycle.
